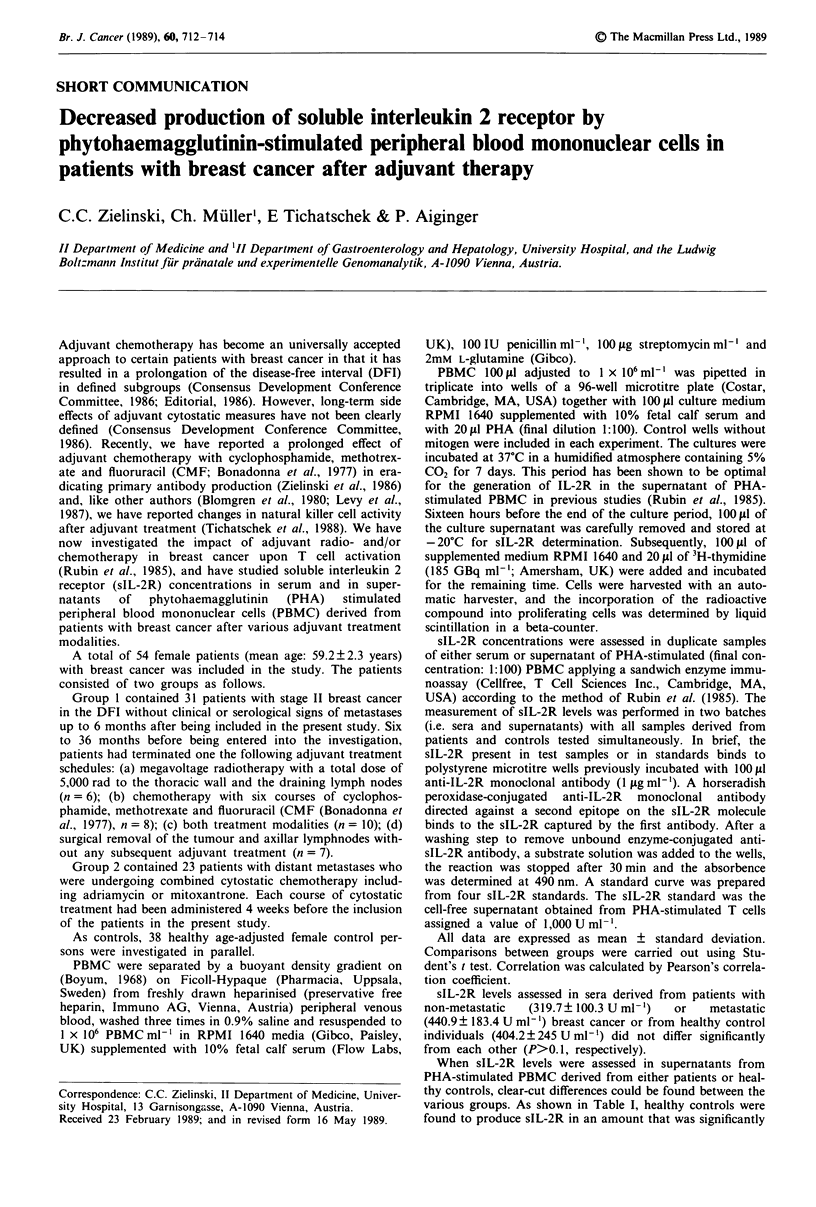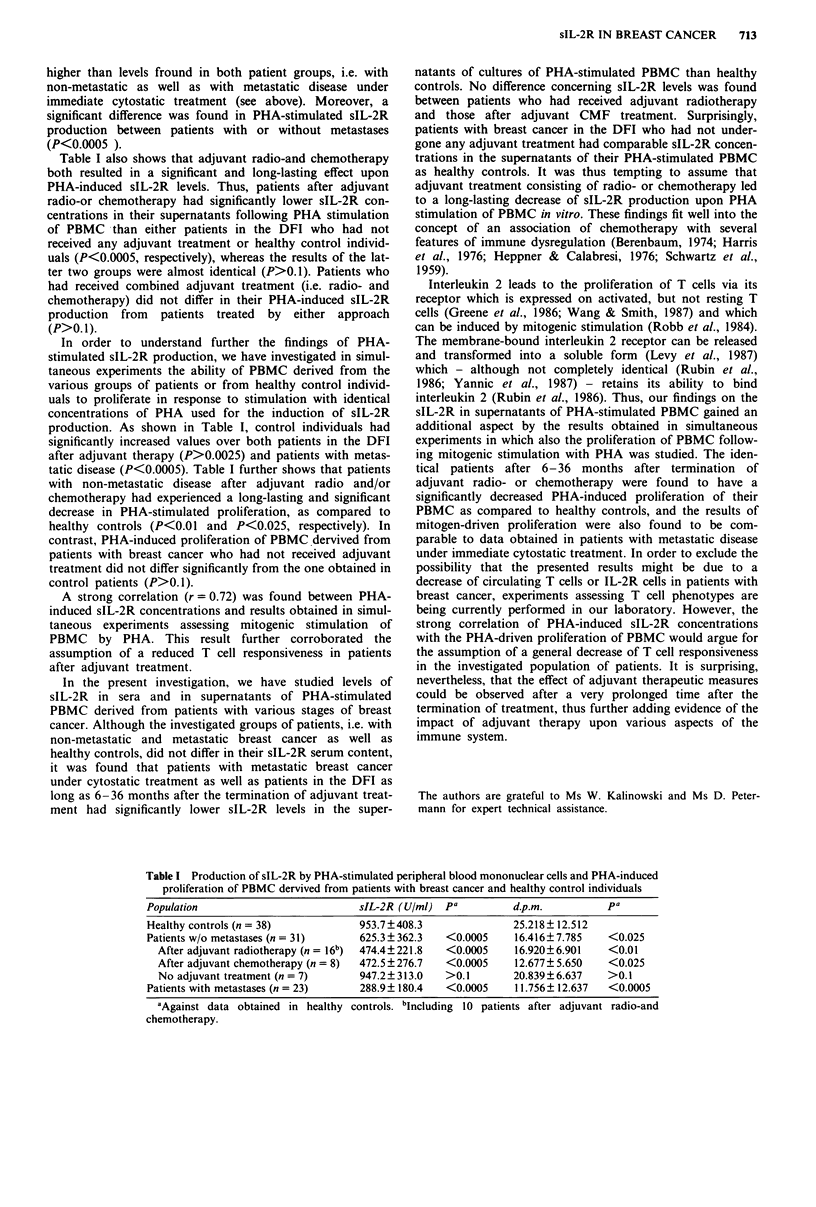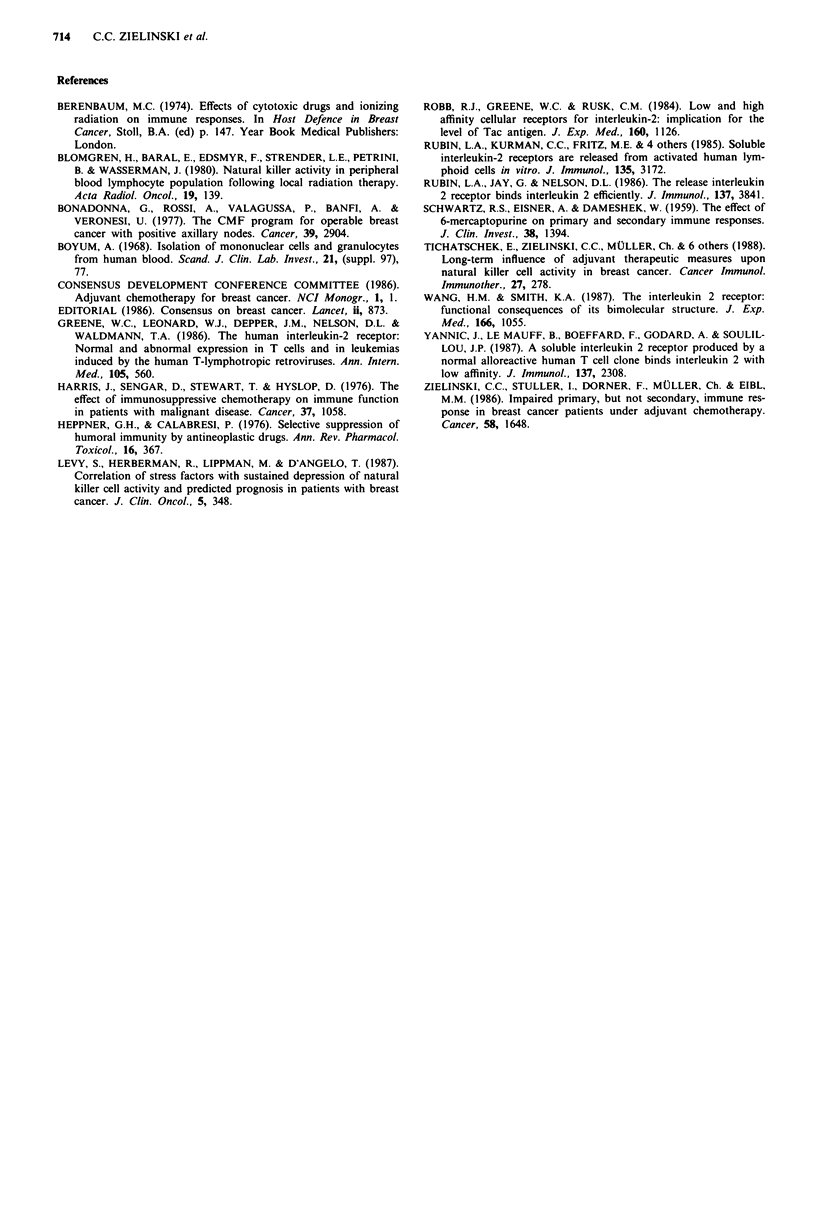# Decreased production of soluble interleukin 2 receptor by phytohaemagglutinin-stimulated peripheral blood mononuclear cells in patients with breast cancer after adjuvant therapy.

**DOI:** 10.1038/bjc.1989.345

**Published:** 1989-11

**Authors:** C. C. Zielinski, C. MÃ¼ller, E. Tichatschek, P. Aiginger

**Affiliations:** II Department of Medicine, University Hospital, Vienna, Austria.


Br. J. Cancer (1989), 60, 712–714 © The Macmillan Press Ltd., 1989

SHORT COMMUNICATION

### Decreased production of soluble interleukin 2 receptor by phytohaemagglutinin-stimulated peripheral blood mononuclear cells in patients with breast cancer after adjuvant therapy

C.C. Zielinski, Ch. Müller[1], E Tichatschek & P. Aiginger

*II Department of Medicine and [1]II Department of Gastroenterology and Hepatology, University Hospital, and the Ludwig Boltzmann Institut für pränatale und experimentelle Genomanalytik, A-1090 Vienna, Austria.*

Adjuvant chemotherapy has become an universally accepted approach to certain patients with breast cancer in that it has resulted in a prolongation of the disease-free interval (DFI) in defined subgroups (Consensus Development Conference Committee, 1986; Editorial, 1986). However, long-term side effects of adjuvant cytostatic measures have not been clearly defined (Consensus Development Conference Committee, 1986). Recently, we have reported a prolonged effect of adjuvant chemotherapy with cyclophosphamide, methotrexate and fluoruracil (CMF; Bonadonna *et al.*, 1977) in eradicating primary antibody production (Zielinski *et al.*, 1986) and, like other authors (Blomgren *et al.*, 1980; Levy *et al.*, 1987), we have reported changes in natural killer cell activity after adjuvant treatment (Tichatschek *et al.*, 1988). We have now investigated the impact of adjuvant radio- and/or chemotherapy in breast cancer upon T cell activation (Rubin *et al.*, 1985), and have studied soluble interleukin 2 receptor (sIL-2R) concentrations in serum and in supernatants of phytohaemagglutinin (PHA) stimulated peripheral blood mononuclear cells (PBMC) derived from patients with breast cancer after various adjuvant treatment modalities.

A total of 54 female patients (mean age: 59.2±2.3 years) with breast cancer was included in the study. The patients consisted of two groups as follows.

Group 1 contained 31 patients with stage II breast cancer in the DFI without clinical or serological signs of metastases up to 6 months after being included in the present study. Six to 36 months before being entered into the investigation, patients had terminated one the following adjuvant treatment schedules: (a) megavoltage radiotherapy with a total dose of 5,000 rad to the thoracic wall and the draining lymph nodes ($n = 6$); (b) chemotherapy with six courses of cyclophosphamide, methotrexate and fluoruracil (CMF (Bonadonna *et al.*, 1977), $n = 8$); (c) both treatment modalities ($n = 10$); (d) surgical removal of the tumour and axillar lymphnodes without any subsequent adjuvant treatment ($n = 7$).

Group 2 contained 23 patients with distant metastases who were undergoing combined cytostatic chemotherapy including adriamycin or mitoxantrone. Each course of cytostatic treatment had been administered 4 weeks before the inclusion of the patients in the present study.

As controls, 38 healthy age-adjusted female control persons were investigated in parallel.

PBMC were separated by a buoyant density gradient on (Boyum, 1968) on Ficoll-Hypaque (Pharmacia, Uppsala, Sweden) from freshly drawn heparinised (preservative free heparin, Immuno AG, Vienna, Austria) peripheral venous blood, washed three times in 0.9% saline and resuspended to $1 \times 10^6$ PBMC $ml^{-1}$ in RPMI 1640 media (Gibco, Paisley, UK) supplemented with 10% fetal calf serum (Flow Labs, UK), 100 IU penicillin $ml^{-1}$, 100 µg streptomycin $ml^{-1}$ and 2mM L-glutamine (Gibco).

PBMC 100 µl adjusted to $1 \times 10^6 ml^{-1}$ was pipetted in triplicate into wells of a 96-well microtitre plate (Costar, Cambridge, MA, USA) together with 100 µl culture medium RPMI 1640 supplemented with 10% fetal calf serum and with 20 µl PHA (final dilution 1:100). Control wells without mitogen were included in each experiment. The cultures were incubated at 37°C in a humidified atmosphere containing 5% $CO_2$ for 7 days. This period has been shown to be optimal for the generation of IL-2R in the supernatant of PHA-stimulated PBMC in previous studies (Rubin *et al.*, 1985). Sixteen hours before the end of the culture period, 100 µl of the culture supernatant was carefully removed and stored at −20°C for sIL-2R determination. Subsequently, 100 µl of supplemented medium RPMI 1640 and 20 µl of $^3$H-thymidine (185 GBq $ml^{-1}$; Amersham, UK) were added and incubated for the remaining time. Cells were harvested with an automatic harvester, and the incorporation of the radioactive compound into proliferating cells was determined by liquid scintillation in a beta-counter.

sIL-2R concentrations were assessed in duplicate samples of either serum or supernatant of PHA-stimulated (final concentration: 1:100) PBMC applying a sandwich enzyme immunoassay (Cellfree, T Cell Sciences Inc., Cambridge, MA, USA) according to the method of Rubin *et al.* (1985). The measurement of sIL-2R levels was performed in two batches (i.e. sera and supernatants) with all samples derived from patients and controls tested simultaneously. In brief, the sIL-2R present in test samples or in standards binds to polystyrene microtitre wells previously incubated with 100 µl anti-IL-2R monoclonal antibody (1 µg $ml^{-1}$). A horseradish peroxidase-conjugated anti-IL-2R monoclonal antibody directed against a second epitope on the sIL-2R molecule binds to the sIL-2R captured by the first antibody. After a washing step to remove unbound enzyme-conjugated anti-sIL-2R antibody, a substrate solution was added to the wells, the reaction was stopped after 30 min and the absorbence was determined at 490 nm. A standard curve was prepared from four sIL-2R standards. The sIL-2R standard was the cell-free supernatant obtained from PHA-stimulated T cells assigned a value of 1,000 U $ml^{-1}$.

All data are expressed as mean ± standard deviation. Comparisons between groups were carried out using Student's *t* test. Correlation was calculated by Pearson's correlation coefficient.

sIL-2R levels assessed in sera derived from patients with non-metastatic (319.7±100.3 U $ml^{-1}$) or metastatic (440.9±183.4 U $ml^{-1}$) breast cancer or from healthy control individuals (404.2±245 U $ml^{-1}$) did not differ significantly from each other ($P > 0.1$, respectively).

When sIL-2R levels were assessed in supernatants from PHA-stimulated PBMC derived from either patients or healthy controls, clear-cut differences could be found between the various groups. As shown in Table I, healthy controls were found to produce sIL-2R in an amount that was significantly

Correspondence: C.C. Zielinski, II Department of Medicine, University Hospital, 13 Garnisongasse, A-1090 Vienna, Austria.
Received 23 February 1989; and in revised form 16 May 1989.

higher than levels fround in both patient groups, i.e. with non-metastatic as well as with metastatic disease under immediate cytostatic treatment (see above). Moreover, a significant difference was found in PHA-stimulated sIL-2R production between patients with or without metastases ($P<0.0005$ ).

Table I also shows that adjuvant radio-and chemotherapy both resulted in a significant and long-lasting effect upon PHA-induced sIL-2R levels. Thus, patients after adjuvant radio-or chemotherapy had significantly lower sIL-2R concentrations in their supernatants following PHA stimulation of PBMC than either patients in the DFI who had not received any adjuvant treatment or healthy control individuals ($P<0.0005$, respectively), whereas the results of the latter two groups were almost identical ($P>0.1$). Patients who had received combined adjuvant treatment (i.e. radio- and chemotherapy) did not differ in their PHA-induced sIL-2R production from patients treated by either approach ($P>0.1$).

In order to understand further the findings of PHA-stimulated sIL-2R production, we have investigated in simultaneous experiments the ability of PBMC derived from the various groups of patients or from healthy control individuals to proliferate in response to stimulation with identical concentrations of PHA used for the induction of sIL-2R production. As shown in Table I, control individuals had significantly increased values over both patients in the DFI after adjuvant therapy ($P>0.0025$) and patients with metastatic disease ($P<0.0005$). Table I further shows that patients with non-metastatic disease after adjuvant radio and/or chemotherapy had experienced a long-lasting and significant decrease in PHA-stimulated proliferation, as compared to healthy controls ($P<0.01$ and $P<0.025$, respectively). In contrast, PHA-induced proliferation of PBMC dervived from patients with breast cancer who had not received adjuvant treatment did not differ significantly from the one obtained in control patients ($P>0.1$).

A strong correlation ($r = 0.72$) was found between PHA-induced sIL-2R concentrations and results obtained in simultaneous experiments assessing mitogenic stimulation of PBMC by PHA. This result further corroborated the assumption of a reduced T cell responsiveness in patients after adjuvant treatment.

In the present investigation, we have studied levels of sIL-2R in sera and in supernatants of PHA-stimulated PBMC derived from patients with various stages of breast cancer. Although the investigated groups of patients, i.e. with non-metastatic and metastatic breast cancer as well as healthy controls, did not differ in their sIL-2R serum content, it was found that patients with metastatic breast cancer under cytostatic treatment as well as patients in the DFI as long as 6–36 months after the termination of adjuvant treatment had significantly lower sIL-2R levels in the supernatants of cultures of PHA-stimulated PBMC than healthy controls. No difference concerning sIL-2R levels was found between patients who had received adjuvant radiotherapy and those after adjuvant CMF treatment. Surprisingly, patients with breast cancer in the DFI who had not undergone any adjuvant treatment had comparable sIL-2R concentrations in the supernatants of their PHA-stimulated PBMC as healthy controls. It was thus tempting to assume that adjuvant treatment consisting of radio- or chemotherapy led to a long-lasting decrease of sIL-2R production upon PHA stimulation of PBMC *in vitro*. These findings fit well into the concept of an association of chemotherapy with several features of immune dysregulation (Berenbaum, 1974; Harris *et al*., 1976; Heppner & Calabresi, 1976; Schwartz *et al*., 1959).

Interleukin 2 leads to the proliferation of T cells via its receptor which is expressed on activated, but not resting T cells (Greene *et al*., 1986; Wang & Smith, 1987) and which can be induced by mitogenic stimulation (Robb *et al*., 1984). The membrane-bound interleukin 2 receptor can be released and transformed into a soluble form (Levy *et al*., 1987) which – although not completely identical (Rubin *et al*., 1986; Yannic *et al*., 1987) – retains its ability to bind interleukin 2 (Rubin *et al*., 1986). Thus, our findings on the sIL-2R in supernatants of PHA-stimulated PBMC gained an additional aspect by the results obtained in simultaneous experiments in which also the proliferation of PBMC following mitogenic stimulation with PHA was studied. The identical patients after 6–36 months after termination of adjuvant radio- or chemotherapy were found to have a significantly decreased PHA-induced proliferation of their PBMC as compared to healthy controls, and the results of mitogen-driven proliferation were also found to be comparable to data obtained in patients with metastatic disease under immediate cytostatic treatment. In order to exclude the possibility that the presented results might be due to a decrease of circulating T cells or IL-2R cells in patients with breast cancer, experiments assessing T cell phenotypes are being currently performed in our laboratory. However, the strong correlation of PHA-induced sIL-2R concentrations with the PHA-driven proliferation of PBMC would argue for the assumption of a general decrease of T cell responsiveness in the investigated population of patients. It is surprising, nevertheless, that the effect of adjuvant therapeutic measures could be observed after a very prolonged time after the termination of treatment, thus further adding evidence of the impact of adjuvant therapy upon various aspects of the immune system.

The authors are grateful to Ms W. Kalinowski and Ms D. Petermann for expert technical assistance.

**Table I** Production of sIL-2R by PHA-stimulated peripheral blood mononuclear cells and PHA-induced proliferation of PBMC dervived from patients with breast cancer and healthy control individuals

| *Population* | *sIL-2R (U/ml)* | $P^a$ | *d.p.m.* | $P^a$ |
|---|---|---|---|---|
| Healthy controls ($n = 38$) | 953.7±408.3 | | 25.218±12.512 | |
| Patients w/o metastases ($n = 31$) | 625.3±362.3 | <0.0005 | 16.416±7.785 | <0.025 |
| After adjuvant radiotherapy ($n = 16^b$) | 474.4±221.8 | <0.0005 | 16.920±6.901 | <0.01 |
| After adjuvant chemotherapy ($n = 8$) | 472.5±276.7 | <0.0005 | 12.677±5.650 | <0.025 |
| No adjuvant treatment ($n = 7$) | 947.2±313.0 | >0.1 | 20.839±6.637 | >0.1 |
| Patients with metastases ($n = 23$) | 288.9±180.4 | <0.0005 | 11.756±12.637 | <0.0005 |

[a]Against data obtained in healthy controls. [b]Including 10 patients after adjuvant radio-and chemotherapy.

### References

BERENBAUM, M.C. (1974). Effects of cytotoxic drugs and ionizing radiation on immune responses. In *Host Defence in Breast Cancer*, Stoll, B.A. (ed) p. 147. Year Book Medical Publishers: London.

BLOMGREN, H., BARAL, E., EDSMYR, F., STRENDER, L.E., PETRINI, B. & WASSERMAN, J. (1980). Natural killer activity in peripheral blood lymphocyte population following local radiation therapy. *Acta Radiol. Oncol.*, **19**, 139.

BONADONNA, G., ROSSI, A., VALAGUSSA, P., BANFI, A. & VERONESI, U. (1977). The CMF program for operable breast cancer with positive axillary nodes. *Cancer*, **39**, 2904.

BOYUM, A. (1968). Isolation of mononuclear cells and granulocytes from human blood. *Scand. J. Clin. Lab. Invest.*, **21**, (suppl. 97), 77.

CONSENSUS DEVELOPMENT CONFERENCE COMMITTEE (1986). Adjuvant chemotherapy for breast cancer. *NCI Monogr.*, **1**, 1.

EDITORIAL (1986). Consensus on breast cancer. *Lancet*, **ii**, 873.

GREENE, W.C., LEONARD, W.J., DEPPER, J.M., NELSON, D.L. & WALDMANN, T.A. (1986). The human interleukin-2 receptor: Normal and abnormal expression in T cells and in leukemias induced by the human T-lymphotropic retroviruses. *Ann. Intern. Med.*, **105**, 560.

HARRIS, J., SENGAR, D., STEWART, T. & HYSLOP, D. (1976). The effect of immunosuppressive chemotherapy on immune function in patients with malignant disease. *Cancer*, **37**, 1058.

HEPPNER, G.H., & CALABRESI, P. (1976). Selective suppression of humoral immunity by antineoplastic drugs. *Ann. Rev. Pharmacol. Toxicol.*, **16**, 367.

LEVY, S., HERBERMAN, R., LIPPMAN, M. & D'ANGELO, T. (1987). Correlation of stress factors with sustained depression of natural killer cell activity and predicted prognosis in patients with breast cancer. *J. Clin. Oncol.*, **5**, 348.

ROBB, R.J., GREENE, W.C. & RUSK, C.M. (1984). Low and high affinity cellular receptors for interleukin-2: implication for the level of Tac antigen. *J. Exp. Med.*, **160**, 1126.

RUBIN, L.A., KURMAN, C.C., FRITZ, M.E. & 4 others (1985). Soluble interleukin-2 receptors are released from activated human lymphoid cells *in vitro*. *J. Immunol.*, **135**, 3172.

RUBIN, L.A., JAY, G. & NELSON, D.L. (1986). The release interleukin 2 receptor binds interleukin 2 efficiently. *J. Immunol.*, **137**, 3841.

SCHWARTZ, R.S., EISNER, A. & DAMESHEK, W. (1959). The effect of 6-mercaptopurine on primary and secondary immune responses. *J. Clin. Invest.*, **38**, 1394.

TICHATSCHEK, E., ZIELINSKI, C.C., MÜLLER, Ch. & 6 others (1988). Long-term influence of adjuvant therapeutic measures upon natural killer cell activity in breast cancer. *Cancer Immunol. Immunother.*, **27**, 278.

WANG, H.M. & SMITH, K.A. (1987). The interleukin 2 receptor: functional consequences of its bimolecular structure. *J. Exp. Med.*, **166**, 1055.

YANNIC, J., LE MAUFF, B., BOEFFARD, F., GODARD, A. & SOULILLOU, J.P. (1987). A soluble interleukin 2 receptor produced by a normal alloreactive human T cell clone binds interleukin 2 with low affinity. *J. Immunol.*, **137**, 2308.

ZIELINSKI, C.C., STULLER, I., DORNER, F., MÜLLER, Ch. & EIBL, M.M. (1986). Impaired primary, but not secondary, immune response in breast cancer patients under adjuvant chemotherapy. *Cancer*, **58**, 1648.